# The Comparative Anatomy of the Temporal Bone, &c.

**Published:** 1838-07

**Authors:** 


ART. X.—*Die vergleichende Osteologie des Schläfenbeins. Zur vereinfachung der herschenden Ansichten bearbeitet.* Von ED. HALLMANN. Mit iv. Kupfertafeln.—*Hannover,* 1837.

*The Comparative Anatomy of the Temporal Bone, and simplification of the prevailing views regarding it.* By ED. HALLMANN.—*Hanover,* 1837.—4to. pp. 130; with iv. Plates.

THIS is a work highly creditable to the industry and knowledge of Mr. Hallmann. His object, in composing it, has been to determine what constitutes the temporal bone in the vertebrate series; to reject the common idea of *a general plan,* which presupposes that a certain bone, or part of it, must necessarily be found in every class; that no piece will be found in one class for which there is not an analogous piece in another. All that is permanent, according to him, in the head is a cranial and a maxillary part. From his observations, he has been led to establish the following law for the development of the head:—" In their development, the skull and jaw proceed in opposite proportions through the series of vertebrate animals from man to fishes." In an appendix, he adduces confirmations of his views from the observations on development by Baer and Rathke, but recants one opinion which he had expressed in the former part of his work, viz. that the petrous bone belongs to the visceral system, and not to the class of cranial bones. An acquaintance, however, with Baer's observations regarding the development of the eye, the ear, and the nose, has led him to view the petrous bone as belonging to the cranium, and the tympanum to the jaw; that the fibrous bones are lateral parts of the cranium, but lateral parts without any proper vertebral body.

